# Supplementary material for: Designing solid-liquid interphases for sodium batteries
Source: Nat Commun. 2017 Oct 12;8:898. doi: 10.1038/s41467-017-00742-x (PMC5638817; doi:10.1038/s41467-017-00742-x)
Supplement: Supplementary file 1 — Supplementary Information [file 41467_2017_742_MOESM1_ESM.pdf]

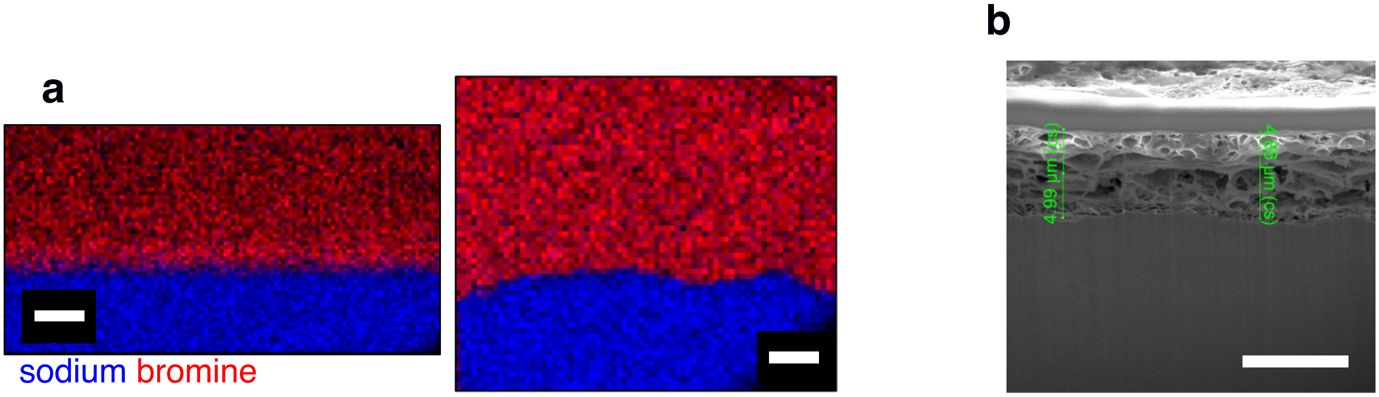

Supplementary Figure 1: Cross-sectional images of NaBr coated sodium and pristine sodium (a) EDX mapping of cross sections of sodium anodes with NaBr surface layers after reacting for 1min (left) and 5mins (right). The cross sections were obtained by cryo-focused ion beam milling; scale bar, 3µm. **(b)** Room temperature FIB-SEM cross-sectional imaging of pristine sodium shows a 5µm thick oxidation layer on the surface; scale bar, 5µm.

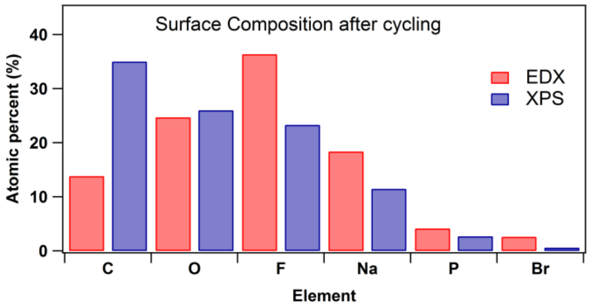

Supplementary Figure 2: **Surface composition of cycled sodium metal with NaBr coating** obtained by two separate methods of EDX and XPS.

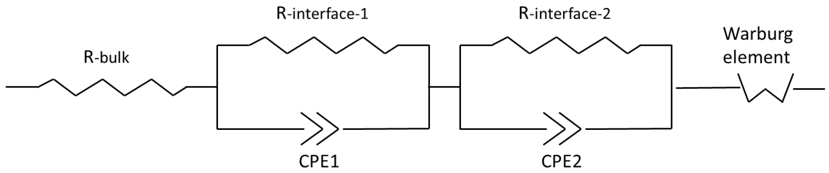

Supplementary Figure 3: **Equivalent circuit model for fitting Nyquist plots of impedance measurements**. Here, R-bulk represents the ion transport in bulk electrolyte. R-interface$_1$ represents interfacial resistance associated with the passivation layer between electrode and electrolyte. R-interface$_2$ denotes the electronic transport in the interface. CPE1, CPE2 represent the constant phase elements. Warburg element stands for solid-state diffusion contribution

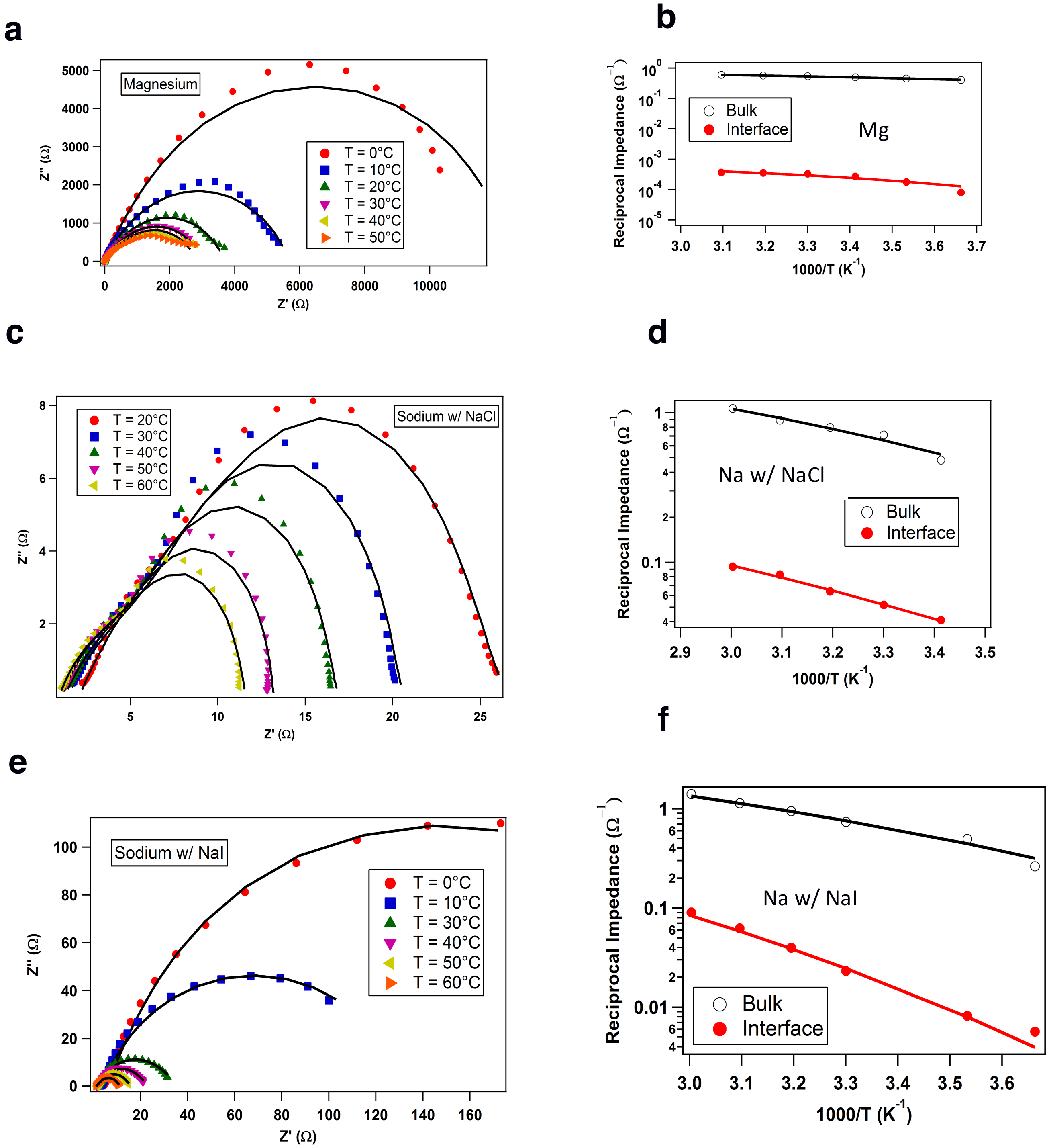

Supplementary Figure 4: **Impedance Spectroscopy for different anodes** Nyquist Plots at various temperatures for **(a)** Mg, **(c)** Na with NaCl, **(e)** Na with NaI. Figures **(b)**, **(d)** and **(f)** represent the temperature dependence of the reciprocal impedances (both bulk and interface) is plotted as a function of Arrhenius Temperature, the lines represent corresponding VFT fits. The labels represent the type of electrode/interface used for the experiment

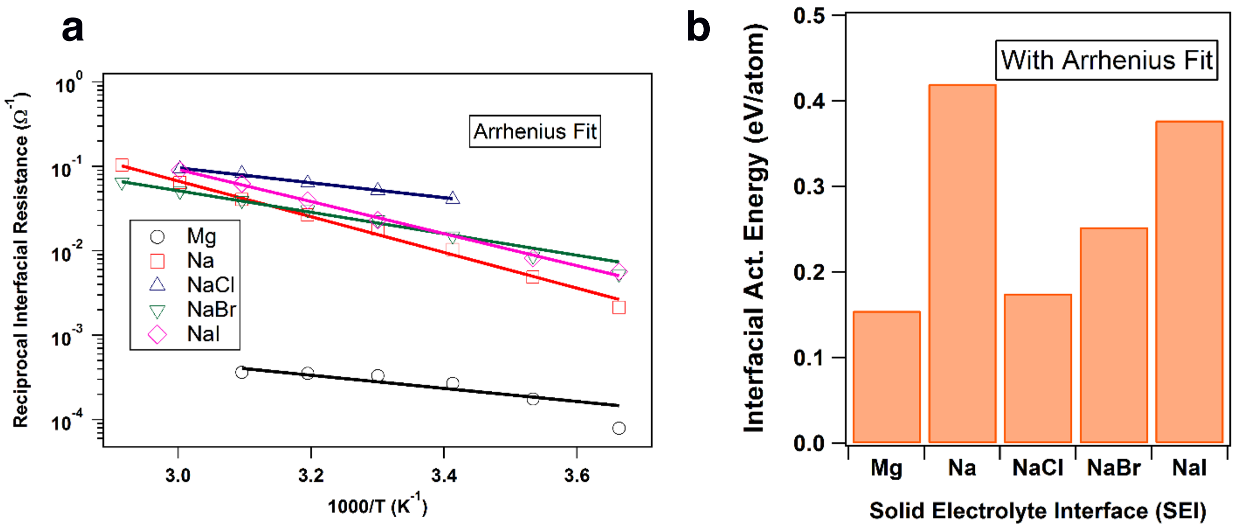

Supplementary Figure 5: Activation energy obtained by Arrhenius Analysis (a) Reciprocal of interfacial resistance plotted as a function of inverse temperature. The fits represent prediction from Arrhenius equation; **(b)** The activation energy is plotted for different interface or metal electrodes, obtained by Arrhenius fitting. Mg, Na represents cells with magnesium and sodium electrodes respectively without any modification, while NaCl, NaBr, NaI represent data for respective halide coated sodium metal.

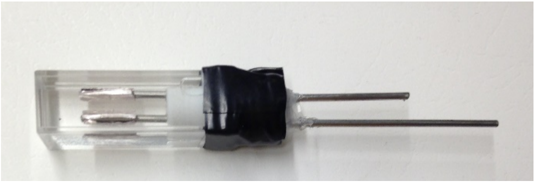

Supplementary Figure 6: **Electrochemical setup for in-situ visualization of sodium electrodeposition** consisting of an airtight cuvette and two rods serving as current collectors for attaching the sodium electrodes. The cap is well sealed with a black tape to ensure that there is no leakage of electrolyte or air-contamination.

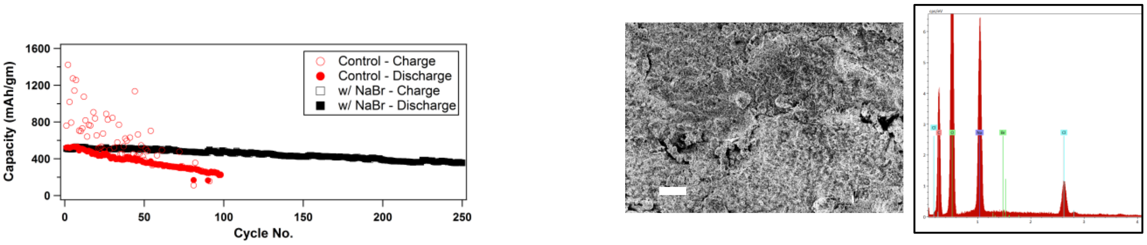

Supplementary Figure 7: Performance and characterization of Na||SPAN cell (a) Charge and discharge capacity as a function of cycle no. for pristine sodium based half-cell and NaBr coated sodium based half-cell comprising of the SPAN cathode. **(b)** SEM image of sodium metal after cycling in Na||SPAN cell with NaBr coating anode; scale bar, 100µm. The adjacent image shows the EDX mapping of the elements of Na anode

Supplementary Table 1: **Parameters of VFT analysis** Bulk impedance represents the ion transport in the electrolyte media; while interfacial impedance indicates the ion transport across electrode-electrolyte interface. The temperature dependent data is fitted to VFT model.

Type of Impedance	Component	Prefactor (A) ('Ω^{-1})	Activation Energy (Ea) (kJ $mole^{-1}$)	Activation Energy (Ea) (eV $atom^{-1}$)	Reference Temp. (To) (K)	Goodness of fit (χ^2)
Bulk	Mg	1.38	1.095	0.011	164.88	6.9×10^{-5}
	Sodium	14.86	3.61	0.037	167.25	1.2×10^{-3}
	NaCl	7.64	2.51	0.026	180	6.4×10^{-3}
	NaBr	6.73	2.14	0.022	197.82	1.9×10^{-3}
	NaI	20.30	3.91	0.041	160	2.1×10^{-2}
Interface	Mg	0.0016	1.32	0.014	211	6.1×10^{-9}
	Sodium	755.81	16.94	0.18	112.84	1.4×10^{-4}
	NaCl	2.44	5.20	0.054	140.43	1.4×10^{-5}
	NaBr	2.70	5.48	0.057	166.40	5.4×10^{-6}
	NaI	37.42	9.14	0.095	152.99	2.0×10^{-5}
